# Rhythm generation by the pre-Bötzinger Complex in medullary slice and island preparations: Effects of adenosine A_1 _receptor activation

**DOI:** 10.1186/1471-2202-9-95

**Published:** 2008-10-01

**Authors:** Richard J VanDam, Edward J Shields, Jonathan D Kelty

**Affiliations:** 1 Department of Biology, Central Michigan University, Mount Pleasant, MI 48858, USA

## Abstract

**Background:**

The pre-Bötzinger complex (preBötC) is a central pattern generator within the ventrolateral medulla oblongata's ventral respiratory group that is important for the generation of respiratory rhythm. Activation of adenosine A_1 _receptors (A_1_R) depresses preBötC rhythmogenesis. Although it remains unclear whether A_1_R activation is important for organisms in a normal metabolic state, A_1_R activation is important to the response of the preBötC to metabolic stress, such as hypoxia. This study examined mechanisms linking A_1_R activation to depression of preBötC rhythmogenesis in medullary slice and island preparations from neonatal mice.

**Results:**

Converting medullary slices to islands by cutting away much of the medullary tissue adjacent to the preBötC decreased the amplitude of action potential bursts generated by a population of neurons within the preBötC (recorded with an extracellular electrode, and integrated using a hardware integrator), without noticeably affecting burst frequency. The A_1_R agonist N^6^-Cyclopentyladenosine (NCPA) reduced population burst frequency in slices by *ca*. 33% and in islands by *ca*. 30%. As in normal (drug-free) artificial cerebrospinal fluid (aCSF), NCPA decreased burst frequency in slices when GABA_A_ergic or GABA_A_ergic and glycinergic transmission were blocked, and in islands when GABA_A_ergic transmission was antagonized. Converting slices to island preparations decreased synaptic input to inspiratory neurons. NCPA further decreased the frequency of synaptic inputs to neurons in island preparations and lowered the input resistance of inspiratory neurons, even when chemical communication between neurons and other cells was impeded.

**Conclusion:**

Together these data support the suggestion that depression of preBötC activity by A_1_R activation involves both decreased neuronal excitability and diminished inter-neuronal communication.

## Background

The pre-Bötzinger complex (preBötC) within the medulla oblongata's ventral respiratory group (VRG) contains a network of neurons important for the generation of ventilatory (inspiratory) rhythmogenesis [1,2]. Even within a 1/2-mm thick transverse slice of medulla the preBötC produces rhythmic bursts of neuronal activity that resemble various patterns of inspiration such as eupneic inspiration, gasps, and sighs [2-7]. Modulation of preBötC rhythmogenesis represents a central focus of research into this region's function. Within transverse medullary slice preparations from neonatal mice, preBötC rhythmogenesis and pattern formation are thought to result from the activity of a heterogeneous population of interneurons, which includes a variety of intrinsically-bursting pacemaker neurons as well as a variety of follower neurons [1,7-10]. Accordingly, modulation of preBötC rhythmogenesis likely involves regulation of multiple aspects of network function, including the modulation of membrane properties and of synaptic interactions [6,11-13].

Adenosine is an important modulator of neuronal network function throughout the CNS [14-17]. For instance, antagonizing adenosine A_1 _receptors (A_1_R) inhibits hypoxic depression of synaptic transmission between hippocampal neurons [15]. Adenosine and A_1_R agonists tend to depress respiratory rhythmogenesis in a variety of neonatal mammals. This finding holds true at the level of the whole organism as well as for *in vitro *preparations containing the preBötC [18-23]. For instance, activation of A_1_R depresses inspiration-related network activity recorded from hypoglossal (XII) nerve rootlets of brainstem-spinal cord preparations obtained from embryonic and neonatal rats, as well as within medullary slice preparations from neonatal mice [22-24]. Depression of respiratory rhythmogenesis by A_1_R may be mediated by its effects on membrane properties, such as increasing conductance of leak K^+ ^channels in preBötC neurons [24]. Network-level depression by A_1_R may also involve reduced synaptic release. Activation of A_1_R pre-synaptically suppresses evoked glutamatergic EPSCs [16] and glycinergic IPSCS [25] in hypoglossal neurons by roughly 42 and 72%, respectively.

Although its role or roles appear to evolve through ontogeny, fast inhibitory synaptic transmission is important in the functioning of central respiration-related networks in mammals ranging from neonatal through adult. In adult mammals inhibitory synaptic transmission appears to be necessary for respiratory rhythmogenesis. For instance, in adult cats antagonism of glycine receptors can block preBötC rhythmogenesis, and injection of the GABA_A _antagonist bicuculline into the preBötC slows respiratory rhythm and induces apneusis [26]. By contrast, the glycine receptor antagonist strychnine injected into the preBötC of adult rats is ineffective in altering phrenic nerve discharge [27]. Within *in situ *preparations from juvenile rats blocking glycinergic transmission can contribute to changing burst shape from incrementing to decrementing [28]. Antagonizing GABA_A_-ergic and glycinergic transmission increases the frequency respiration-related bursts of neuronal activity generated by brainstem slices from neonatal mice [29-31] and brainstem spinal cord preparations [29]. Moreover, blocking GABA_A _and glycine receptors increases the amplitude of integrated bursts generated by brainstem slices [30], increases the area of integrated bursts produced by brainstem spinal cord preparations and medullary slice preparations [29], increases the excitability in the medullary slice preparation [31], and allows medullary slice preparations to generate rhythmic bursts when bathed in 3 mM K^+^, rather than 8 mM K^+ ^[32]. Activation of GABA_A _receptors in brainstem spinal cord and slice preparations from embryonic (on or after embryonic day 19) and neonatal rats slows respiration-related bursting when the preparations are bathed in artificial cerebrospinal fluid (aCSF) containing 3 mM K^+^, but increases burst frequency when the preparation are bathed in aCSF with elevated extracellular K^+ ^(9 mM) [33]. Thus, within *in vitro *preparations from neonatal mice and rats, inhibitory synaptic transmission affects the pattern of respiration-related output, rather than being required for rhythmogenesis.

The purpose of this study was to examine factors that may contribute to, or interact with, A_1_R-mediated depression of preBötC rhythmogenesis including effects of A_1_R activation on synaptic transmission and membrane properties. Since GABA_A_ergic and glycinergic transmission affect the pattern of pre-BötC output, the effect of A_1_R activation on inputs via these transmitters was examined as proxy for the effects of A_1_R activation on fast chemical transmission in general. Although the pattern of respiration-related bursting in medullary slice preparations from neonatal mice is altered during GABA_A _and glycine receptor antagonism its persistence provides the opportunity to determine whether baseline GABA_A_ergic/Glycinergic transmission and the effects of A_1_R activation interact. That is, when GABA_A_ergic or GAB_A_Aergic and glycinergic transmission are left intact, is the network-level depression observed during A_1_R activation more extensive than when synaptic input is reduced or when GABA_A _or GABA_A _and glycine receptors are extensively antagonized?

Whereas this study used GABA_A_ergic and glycinergic antagonists to extensively block these forms of fast chemical transmission, the effects of less severe reduction in intra- and/or inter-network synaptic transmission on the preBötC's population-level response to A_1_R activation were examined by comparing the actions of the A_1_R agonist N^6^-Cyclopentyladenosine (NCPA) in medullary slice preparations and preBötC island preparations. Reducing the medullary slice to an island preparation removes regions including the inferior olivary complex (IO), spinal trigeminal tract (SP5), nucleus tractus solitarius (NTS), medullary raphe, XII nucleus, facial nucleus, and contralateral preBötC [34]. As with the medullary slice preparation, portions of the VRG abutting the preBötC (along the rostro-caudal axis) remain in the island. Conversion to the island preparation severs the axons of many neurons projecting to the preBötC, and may reduce intra-network communication, with the net effect being a reduction in the amount of input received by preBötC neurons as show in the results section below [*c.f*. [34]]. Thus, many or most of the currents observed in preBötC neurons within the island preparation are likely evoked by transmitters released from preBötC neurons, or from other VRG neurons.

The data presented herein demonstrate that A_1_R activation depresses preBötC rhythmogenesis similarly in otherwise untreated slice preparations, when synaptic input is reduced by converting the slice to an island, and in slice preparations within which GABA_A_ergic or GABA_A_ergic and glycinergic receptors were antagonized. This study further demonstrates that during its depression of preBötC rhythmogenesis A_1_R activation decreases synaptic input to preBötC neurons and alters resting membrane properties in a manner consistent with decreased neuronal excitability. Together these findings support the notion that although the synaptic currents/potentials evoked by baseline levels of GABA and glycine may integrate with the modulatory effects of A_1_R activation, their contribution is too small to be noticed at the level of population level recordings. Rather the effects of A_1_R activation on membrane properties and/or synaptic release are sufficient to cause substantial depression of the preBötC.

## Results

### Effects of A_1_R agonism/antagonism on preBötC population activity in slice preparations

**Figure 1:**
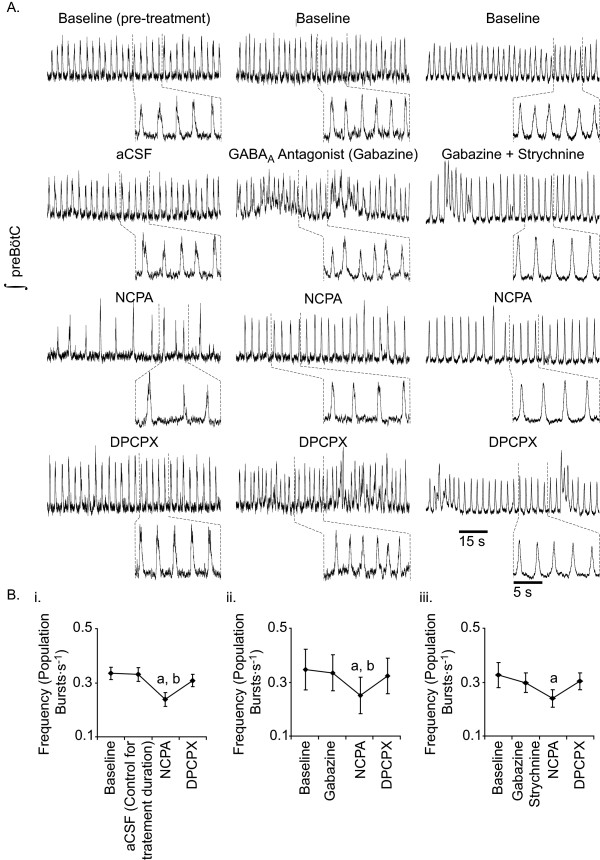
**Effects of A_1_R activation on preBötC rhythmogenesis in medullary slice preparations**. A. Representative effects of bath-applied NCPA (1 μM) alone, NCPA in the presence of gabazine (20 μM), or NCPA in combination with gabazine (20 μM) and strychnine (1 μM). B. Whether applied (i) alone, (ii) with gabazine, or (iii) with gabazine and strychnine, NCPA decreased burst frequency (**a**, value different from baseline at P < 0.05, Tukey post-hoc test; **b**, value different from step 2 of treatment – either continued aCSF, application of gabazine, or application of gabazine and strychnine – at P < 0.05, Tukey post-hoc test).

Pharmacological manipulation of A_1_R affected the generation of population bursts by the preBötC (Repeated Measures ANOVA P < 0.001; Table 1). The A_1_R agonist NCPA (1 μM) decreased population burst frequency by 27.3% (P < 0.001; Fig. 1A, B. Subsequent addition of the A_1_R antagonist 1,3-Dipropyl-8-cyclopentylxanthine (DPCPX; 1 μM, n = 12) returned population bursting to essentially baseline frequency (P < 0.001 vs. NCPA; Fig. 1A, B). The amplitude of population bursts generated during NCPA treatment was statistically indistinguishable from that of bursts generated during baseline recording (Repeated Measures ANOVA, P > 0.1; Table 1).

**Table 1:** Effects of A_1_R activation on population burst parameters

			Slice			Island	
							
Treatment Group	Stage of Experiment	n	Frequency (bursts·sec^-1^)	Amplitude	n	Frequency (bursts·sec^-1^)	Amplitude
aCSF control	1. Baseline	12	0.33 ± 0.02	2.01 ± 0.24	9	0.37 ± 0.04	1.39 ± 0.25
	**2. aCSF**		0.33 ± 0.03	2.03 ± 0.25		0.36 ± 0.04	1.50 ± 0.31
	3. NCPA		0.24 ± 0.03	2.13 ± 0.24		0.26 ± 0.04^a, b^	1.57 ± 0.40
	4. DPCPX		0.31 ± 0.02	1.81 ± 0.22		0.37 ± 0.03	1.48 ± 0.44
							
Bicuculline	1. Baseline	11	0.36 ± 0.03	1.65 ± 0.23	7	0.44 ± 0.06	1.13 ± 0.19
	**2. Bicuculline**		0.33 ± 0.02	2.27 ± 0.35^a^		0.43 ± 0.05	1.77 ± 0.29^a^
	3. NCPA		0.22 ± 0.03^a, b^	2.33 ± 0.35^a^		0.41 ± 0.05	1.55 ± 0.21^a^
	4. DPCPX		0.31 ± 0.03	1.93 ± 0.30		0.48 ± 0.07	1.81 ± 0.37^a^
							
Gabazine	1. Baseline	7	0.34 ± 0.07	3.10 ± 1.14	6	0.27 ± 0.04	0.89 ± 0.10
	**2. Gabazine**		0.33 ± 0.06	3.48 ± 1.24		0.29 ± 0.03	0.86 ± 0.10
	3. NCPA		0.25 ± 0.07^a, b^	2.56 ± 0.89		0.18 ± 0.05^a, b^	0.89 ± 0.15
	4. DPCPX		0.32 ± 0.06	3.37 ± 1.11		0.30 ± 0.05	0.76 ± 0.09
							
Gabazine & Strychnine	1. Baseline	8	0.33 ± 0.05	0.97 ± 0.16	6	0.40 ± 0.06	1.15 ± 0.16
	**2. Gabazine + Strychnine**		0.30 ± 0.04	1.40 ± 0.20		0.40 ± 0.05	1.46 ± 0.23^a^
	3. NCPA		0.24 ± 0.03^a, b^	1.31 ± 0.23		0.34 ± 0.05	1.51 ± 0.26^a^
	4. DPCPX		0.30 ± 0.03	1.10 ± 0.17		0.38 ± 0.04	1.45 ± 0.26^a^

Slices were superfused with one of four solutions prior to treatment with NCPA (aCSF alone, aCSF with bicuculline, aCSF with gabazine, or aCSF with gabazine and strychnine).

^1^All values presented as mean ± SEM.

^a^Different from baseline (pre-treatment) value at P < 0.05 (Tukey post-hoc tests)

^b^Different from control (aCSF only) at P < 0.05 (Tukey post-hoc tests)

### Effects of A_1_R manipulation on slice rhythmogenesis during GABA_A _receptor antagonism

A second set of experiments determined whether the post-synaptic effects of GABA_A_ergic transmission integrate with (effectively sum with) the effects of A_1_R activation to increase the level of network-level depression of preBötC rhythmogenesis in the medullary slice preparation. Note: an alternative hypothesis could be that A_1_R activation substantially increases extracellular levels of GABA and/or glycine thereby depressing the activity of postsynaptic neurons and thus depressing network activity. However, given that A_1_R activation tends to depress synaptic transmission throughout the nervous system [16,25], such an effect is unlikely. Due to potential non-specific effects of bicuculline on neuronal properties, a subset of experiments used gabazine (20 μM), rather than bicuculline to antagonize GABA_A _receptors. NCPA decreased the overall frequency of population bursts in slices by ~33% during bicuculline treatment (n = 11; P < 0.001; Table 1) and by ~24.2% during gabazine treatment (n = 7; P < 0.05; Fig. 1A, Bii). These changes in frequency were indistinguishable from the ~27.3% decrease observed in slices treated with NCPA alone. As with slices treated with NCPA alone, DPCPX applied during treatment with NCPA and bicuculline returned population burst frequency to a level (0.31 ± 0.03 Hz) statistically indistinguishable from baseline. Similarly, in slices treated with gabazine and NCPA, DPCPX increased population burst frequency to a level indistinguishable from treatment with gabazine alone (Table 1).

Population burst amplitude varied between treatments in slice preparations treated with the GABA_A _receptor antagonist bicuculline (Repeated measures ANOVA, P = 0.002), but not in slices treated with gabazine (Repeated Measures ANOVA, P = 0.815; Table 1). Bicuculline increased the amplitude of population-level bursting (Tukey Post-Hoc Test, P = 0.009; Table 1). Agonism/antagonism of A_1_R during antagonism of GABA_A _receptors with bicuculline or gabazine produced no statistically detectable change in mean burst amplitude (Table 1).

### Effects of A_1_R manipulation on slice rhythmogenesis during GABA_A _and glycine receptor antagonism

**Figure 2:**
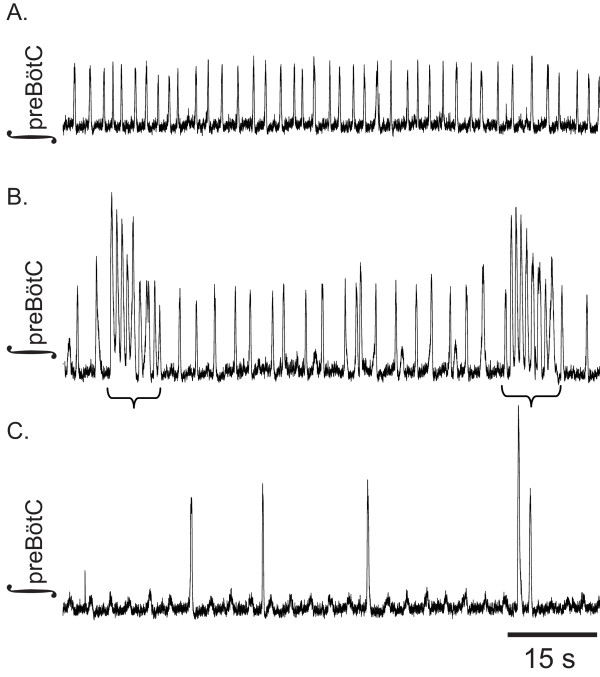
**Block of chloride-mediated inhibition inducesseizure-like activity in medullary slice preparations**. Three sequential sample recordings of integrated preBötC activity from a single medullary slice preparation. A. Activity recorded in recorded in drug free aCSF. B. Gabazine (20 μM) and Strychnine (1 μM) induce seizure-like bursting (brackets) characterized by increased burst frequency and elevated baseline, while slightly decreasing the frequency of population bursts generated between seizure-like bursts. C. Antagonism of A_1_R with NCPA (1 μM) eliminated seizures for this slice and decreased population burst frequency.

In all 8 slices examined, a cocktail of gabazine (20 μM) and strychnine (1 μM) induced seizure-like bursting at 1.36 ± 0.22 siezures·min^-1 ^(Fig. 2. During subsequent agonism of A_1_R with NCPA seizures occurred at 0.71 ± 0.47 seizures·min^-1 ^(P > 0.05, Tukey Test). Blockade of Cl^-^-mediated transmission did not discernibly affect the overall frequency of population bursting. Even with GABA_A_ergic and glycinergic transmission antagonized, NCPA decreased the frequency of population bursts relative to baseline (Fig. 1A, B; P = 0.002, Tukey Test). This change in frequency was indistinguishable from the ~27.2% decrease observed in slices treated with NCPA alone. Although a repeated measures ANOVA indicated significant overall variation in amplitude between all treatments (in slices treated with gabazine and strychnine; P = 0.044), Tukey post-hoc tests demonstrated that none of the individual pairs of treatments were distinguishable from each other (Table 1).

### Effects of A_1_R agonism/antagonism on preBötC population activity in island preparations

**Figure 3:**
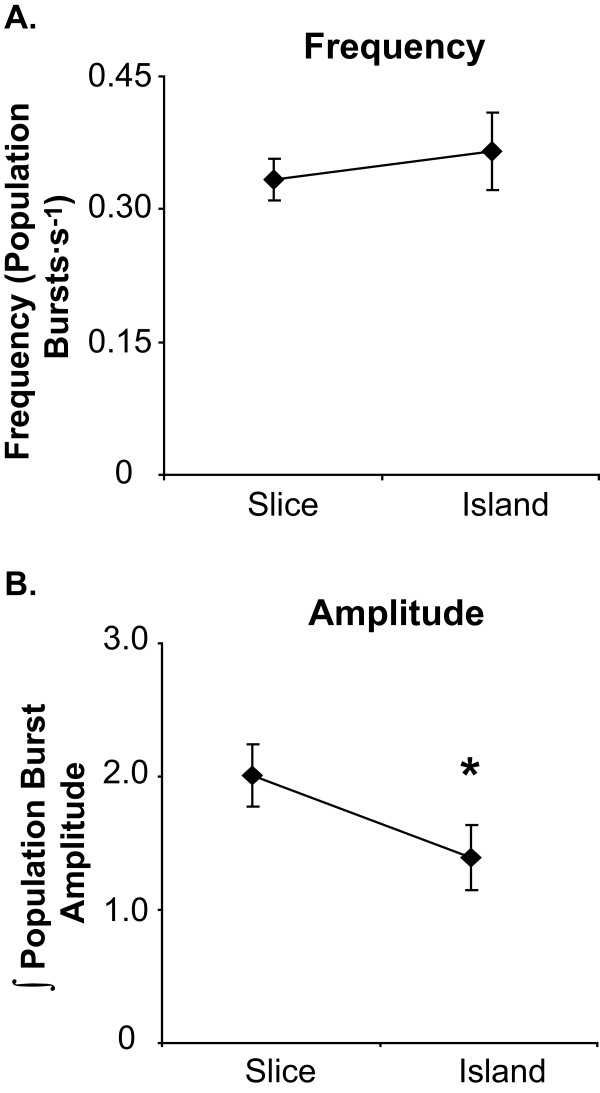
**Effects of slice to island conversion onpopulation burst parameters**. A. The frequency of population bursts generated by the preBötC was unchanged by cutting away regions of the slice preparation adjacent to the preBötC (see text), thereby converting the section to an island preparation (frequency = 0.33 ± 0.02 Hz in slices vs. 0.37 ± 0.04 Hz in islands; n = 13 of each; paired t-test, P = 0.73). B. By contrast, the amplitude of integrated population bursts decreased by 30.1% with conversion of the slice to the island preparation (*, P < 0.05, paired t-test).

**Figure 4:**
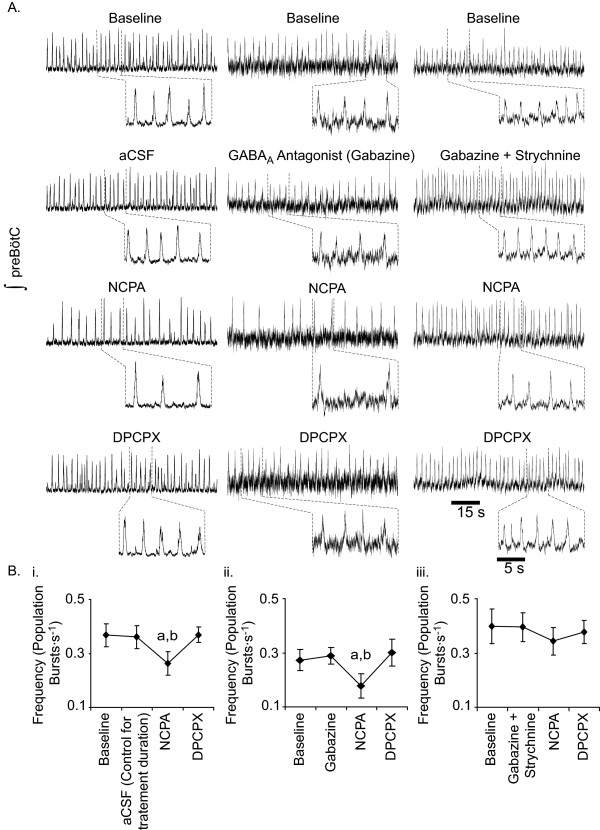
**Effects of A_1_R activation on preBötC rhythmogenesis in island preparations**. A. Representative effects of NCPA (1 μM) alone, with gabazine (20 μM) and in combination with 20 μM gabazine and 1 μM strychnine. B. As with slice preparations, NCPA alone (i) or in combination with gabazine (ii) decreased burst frequency. By contrast to slices, NCPA applied in combination with gabazine and strychnine (iii) failed to affect burst frequency (**a**, value different from baseline at P < 0.05, Tukey post-hoc test; **b**, value different from the second step of treatment – either continued aCSF, application of gabazine, or application of gabazine and strychnine – at P < 0.05, Tukey post-hoc test).

The slices from which island preparations were excised and the islands generated from them produced population bursts at similar frequencies (Fig. 3. However, population burst amplitude decreased with conversion from slice to island preparation (n = 9; P < 0.001; Fig. 3). As with slice preparations, NCPA decreased the frequency of population bursts generated by islands, in this case from 0.37 ± 0.04 to 0.26 ± 0.04 Hz (n = 9; P < 0.001; Table 1). This decrease (~29.8%) was indistinguishable from the 27.3% decrease observed in slices treated with NCPA alone. Subsequent application of DPCPX, in the continued presence of NCPA, increased the frequency of population bursting to a level indistinguishable from baseline (0.37 ± 0.03 Hz; Tukey Test, P < 0.001, vs. NCPA; Fig. 4. Population burst amplitude remained similar between treatments in island preparations (Repeated measures ANOVA, P = 0.547; Table 1).

### Effects of A_1_R manipulation on island rhythmogenesis during GABA_A _receptor antagonism

The frequency of population bursts generated by island preparations remained near baseline levels following addition of bicuculline (Tukey Test, n = 7, P = 0.981; Table 1). By contrast to slices, NCPA applied to islands in the presence of bicuculline failed to change population burst frequency relative to baseline (Tukey test, n = 7, P = 0.493). As in slice preparations, bicuculline increased population burst amplitude in island preparations (Tukey test, P = 0.027; Table 1), which effectively remained unchanged during subsequent treatment with NCPA (Tukey test, P = 0.695; Table 1).

By contrast to its effects in the presence of bicuculline, NCPA applied in the presence of gabazine decreased the frequency of population bursting in island preparations (Tukey test, n = 6, P < 0.001; Fig. 4). Moreover, A_1_R activation reduced burst frequency to a greater extent in gabazine-treated islands than in gabazine-treated slices causing a ~37.9% decrease in island burst frequency compared to a 24.2% decrease in gabazine treated slices (t-test, P = 0.0002). As with bicuculline, gabazine caused no detectable change in population burst frequency in island preparations (Fig. 4A, Bii). Unlike bicuculline, gabazine caused no discernible change in the amplitude of population bursts in island preparations. However, in the presence of gabazine burst amplitude remained relatively constant during treatment with NCPA (Table 1).

### Effects of A_1_R manipulation on island rhythmogenesis during GABA_A _and glycine receptor antagonism

Population burst frequency was seemingly unaffected by combined GABA_A _and glycine receptor antagonism, with islands generating population bursts at 0.40 ± 0.09 Hz under baseline conditions and at 0.40 ± 0.08 Hz in the presence of gabazine and strychnine (n = 6; Fig. 4A, Biii). By contrast to medullary slices, the combination of gabazine and strychnine evoked no seizures in island preparations. In the presence of gabazine and strychnine, NCPA failed to cause a statistically detectable change in population burst frequency or amplitude (Repeated measure ANOVA, n = 6, P = 0.16; Table 1).

### Effects of A_1_R activation on synaptic inputs

**Figure 5:**
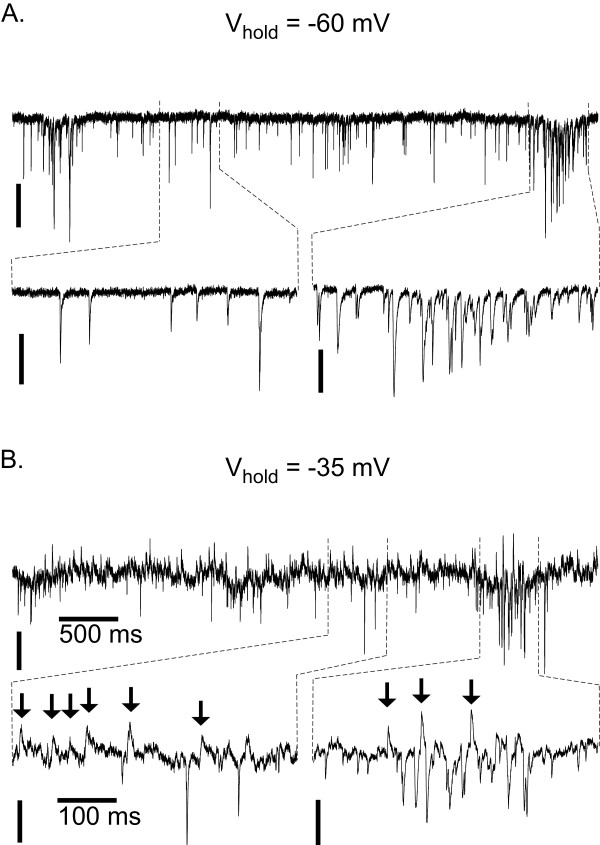
**Synaptic inputs to preBötC neurons**. A. Spontaneous postsynaptic currents from an inspiratory neuron voltage clamped at -60 mV. Note both iPSCs from ePSCs appear as inward currents. B. The same neuron as in A, but voltage clamped at -35 mV. Note that chloride-mediated synaptic currents now appear as outward currents (arrows). Although it is impossible to accurately measure the amplitude or frequency of excitatory or inhibitory inputs during inspiration-related bursts of synaptic input it is possible to distinguish between excitatory and inhibitory inputs during the interburst interval. Vertical scale: 50 pA.

With the solutions used during this study all synaptic inputs appeared to evoke inward currents in neurons voltage-clamped at -60 mV (Fig. 5A. Thus, to discriminate between inhibitory inputs and excitatory inputs inspiratory neurons were voltage clamped at -35 mV, at which Cl^-^-mediated currents appeared as outward events and excitatory currents appeared as inward events (Fig. 5B). During population bursts inhibitory and excitatory inputs occurred at a high enough frequency that the resultant summation/interference prohibited evaluation of synaptic inputs during this period. Rather, sEPSC and sIPSC frequencies were evaluated during the period between population bursts.

**Figure 6:**
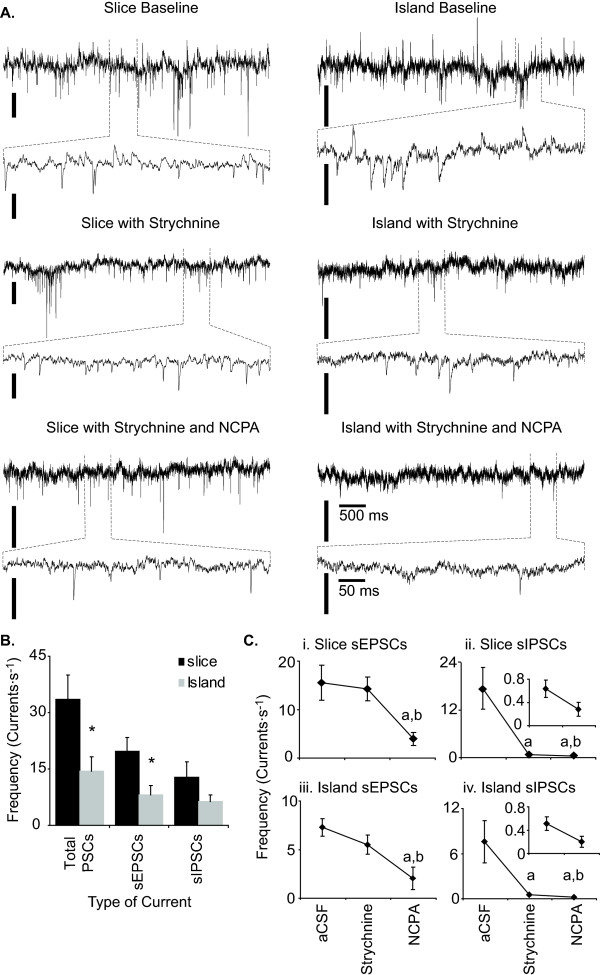
**Manipulation of A_1_R affects synaptic input to preBötC neurons**. A. Representative current traces from slice (left) and island preparations (right). Vertical scale: 50 pA. B. Converting slices to island preparations reduced the frequency of total sPSCs and sEPSCs evoked in preBötC neurons (*P < 0.05, Two-sample t-test). This trend appeared to hold true for sIPSCs but did not attain statistical significance. C. NCPA (1 μM) decreased the frequency of sEPSCs and sIPSCs received by preBötC neurons within slice (i, ii) and island preparations (iii, iv) in the presence of strychnine (1 μM). The insets in (ii) and (iv) are magnified views of sIPSC frequencies in the presence of strychnine. Letters above columns show difference from mean frequency under baseline (**a**, P < 0.05) conditions and in the presence of strychnine (**b**, P < 0.05).

During baseline recording, inspiration-related neurons (those receiving increased excitatory input during the population burst) within slice preparations received sEPSCs at 15.6 ± 3.6 Hz, and sIPSCs at 17.5 ± 5.3 Hz (Fig. 6B. Strychnine eliminated almost all sIPSCs, reducing their frequency to 0.6 ± 0.1 Hz (n = 8; P = 0.001), without noticeably affecting sEPSC frequency (Fig. 6A, C). NCPA further decreased the frequency of sIPSCs to 0.3 ± 0.1 Hz (n = 8; P = 0.001). In each of the 8 neurons treated sequentially with strychnine and then NCPA, subsequent treatment with bicuculline eliminated all remaining sIPSCs. As with sIPSCs, NCPA decreased the frequency of sEPSCs received by inspiratory neurons, in this case from 15.6 ± 3.6 to 4.0 ± 1.4 Hz (n = 8, P = 0.004; Fig. 6C).

Reducing slice preparations to islands reduced the combined frequency of sIPSCs and sEPSCs received by inspiration-related neurons by 59%, (n = 13 of each type of preparation; P = 0.004, Mann-Whitney Rank Sum Test; Fig. 6A, B). Reducing medullary slices to island preparations decreased sEPSC frequency in inspiration-related neurons from 19.8 ± 3.8 (n = 13 slices) to 8.1 ± 2.4 Hz (n = 13 islands; P < 0.001, Mann-Whitney Rank Sum Test). Conversion from medullary slice to island preparation also appeared to reduce sIPSC frequency in inspiration-related neurons (from 17.5 ± 5.3 Hz to 7.6 ± 2.8 Hz). However, this change failed to attain statistical significance (n = 13 of each type or preparation, P = 0.167; Fig. 6B).

Although island preparations received synaptic inputs at lower frequencies than slice preparations, their response to NCPA, in this regard, was much the same. Within island preparations, strychnine reduced sIPSC frequency from 7.6 ± 2.8 to 0.5 ± 0.1 Hz (n = 6, P < 0.001; Fig. 6C). Addition of NCPA further decreased GABA_A_ergic sIPSC frequency to 0.2 ± 0.1 Hz (Mann-Whitney Rank Sum Test, P = 0.041), and decreased sEPSC frequency to 2.0 ± 1.2 Hz (n = 6, Two Sample t-test, P = 0.035).

### Effects of A_1_R activation on membrane properties

Since A_1_R activation is known to affect membrane properties, and in some neurons overall excitability, we examined the effects of NCPA on the R_in_, I_Na _(only without synaptic isolation) and I_Kd _of inspiratory preBötC neurons. In slice and island preparations (n = 10) bathed in normal aCSF, NCPA decreased R_in _from 389.2 ± 130.0 to 287.7 ± 113.3 MΩ (Paired t-test, P < 0.05). After 15 min of recording, the R_in _of one of these neurons increased by 8% and in two others R_in _remained essentially unchanged from baseline (<5% change). Overall, steady state outward currents evoked by voltage steps applied in 10 mV increments between -80 mV and +20 mV were unaffected by NCPA (Paired t-test, P > 0.1). Similarly, in the presence of NCPA voltage steps from -60 mV to -40 mV evoked I_Na _(-4192.2 ± 919.8 pA) similar to that evoked by identical voltage steps under baseline conditions (-5291.2 ± 1387.9 pA; n = 5, P = 0.53).

**Figure 7:**
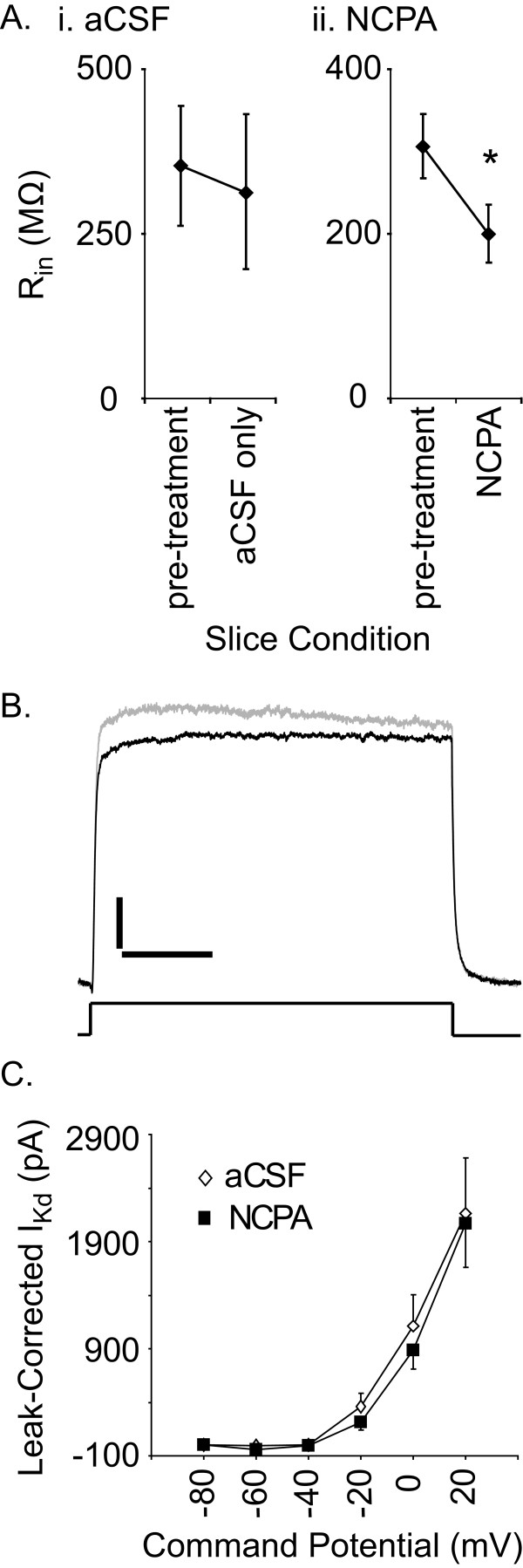
**Effects of A_1_R activation on individual inspiration-related preBötC neurons**. A. NCPA (1 μM) decreased R_in _of synaptically isolated neurons (n = 7; * P < 0.05). B. In this representative pair of whole cell current traces evoked by a voltage step from -60 to 20 mV I_Kd _is similar during recording in normal aCSF (black trace) and in NCPA (grey trace). Vertical scale: 100 pA; time scale: 50 ms. C. Mean current-voltage relationship for inspiratory neurons (n = 5; error bars represent SEM).

To verify that NCPA directly affected A_1_R activation in the cells examined, rather than affecting release of other substances onto the patched cells, we repeated the preceding set of experiments (minus the measurement of I_Na_) in the presence of TTX, Cd^++^, elevated Mg^++ ^and minimal extracellular Ca^++ ^(as close to no Ca^++ ^as possible). Whereas input resistance remained nearly constant in the modified aCSF (15 min control for NCPA treatment period; Fig. 7Ai, addition of NCPA decreased R_in _from 306.9 ± 38.4 to 200.8 ± 34.0 MΩ (n = 7, Paired t-test, P = 0.018). By contrast to its effects on R_in_, NCPA produced no detectable effect on (leak-corrected) I_Kd _(n = 5; Fig. 7B, C).

## Discussion

In preparations representing levels of biological organization from tissue through whole-organism, A_1_R activation depresses neuronal-network activity underlying respiratory rhythmogenesis [23,24]. Although GABA_A_ergic and glycinergic transmission affect the patterning and excitability of the respiratory network within *in vitro *preparations from neonatal mammals, the data presented herein show that the depression of preBötC rhythmogenesis by A_1_R activation is unaffected by reducing the overall level of synaptic input received by preBötC neurons, or by antagonizing GABA_A _or GABA_A _and glycine receptors. Thus, while the postsynaptic currents/potentials caused by GABA_A_/glycine receptor activation may integrate with the modulatory effects of A_1_R activation, the relative contribution of such integration to the combined effect is minor. The intracellular data presented herein are consistent with the notion that A_1_R-mediated depression of network activity may involve modulation of resting membrane properties as well as suppression of synaptic release.

The preBötC receives synaptic input from other regions within the slice preparation. Modulation of at least some of these inputs, such as the contralateral preBötC, can affect the pattern of preBötC output [10,34]. Converting the slice to an island preparation removes the somata of many neurons sending axons to the preBötC. In excised preparations representing a variety of CNS regions, axon terminals severed from their somata continue to release messenger for a period of time after being cut away. In fact, such release may be affected by the application of modulatory substances to the cut terminals [35]. However, converting the slice preparation to the island preparation reduces synaptic input to preBötC neurons (e.g., Fig. 6) suggesting that a large proportion of cut terminals in the island lose much, if not all, of their function shortly after being cut and/or that conversion leads to a decrease in intra-network communication. Activation of A_1_R depresses preBötC rhythmogenesis similarly in slice and island preparations, the latter representing a condition of reduced synaptic transmission. These findings, are consistent with the notion that the modulatory effects of A_1_R activation, although likely to integrate with concurrent synaptic inputs, are greater in their overall effect on preBötC activity than are A_1_R-induced changes in the net synaptic input to preBötC neurons from sources originating outside the preBötC.

Although not required for rhythmogenesis in the neonatal respiratory network GABA_A_ergic and glycinergic transmission affect the pattern of respiratory network output [28-31,33,36] and blocking these forms of communication increases network excitability [31,32]. Here antagonizing GABA_A _and glycine receptors induced seizure-like bursting in slice preparations. Within the island preparation antagonism of GABA_A _and glycine receptors appeared to synchronize preBötC bursting, increasing burst amplitude [34]. Interestingly, this apparent synchronization during GABA_A _and glycine receptor antagonism occurred in islands from both very young (postnatal day 0- postnatal day 4), and older (postnatal day 4- postnatal day 7) mice. Although the Cl^- ^equilibrium potential of preBötC neurons shifts from depolarizing to hyperpolarizing at around embryonic day 19 [33] for mice, this shift is not apparent until after postnatal day 2 [30] when medullary slice preparations are bathed in aCSF containing elevated K^+ ^[30,33].

As noted above, GABA_A_ergic and glycinergic transmission affect respiratory network excitability and the pattern of respiratory network bursting. Accordingly, this study determined whether the blocking the postsynaptic currents/potentials evoked by activation of GABA_A _and glycine receptors may alter the extent of network depression observed during A_1_R activation. That is, do the postsynaptic potentials evoked by GABA_A_, or GABA_A _and glycine receptor activation integrate synergistically with the depressive modulatory effects of A_1_R activation? The lack of any noticeable difference in the response to A_1_R activation between slices in standard aCSF from those in aCSF with gabazine or with gabazine and strychnine suggests that the postsynaptic currents/potentials evoked by baseline GABA_A_/glycine receptor activation contribute little, if at all, to a potential combined effect.

In the medullary slice preparations used for this study baseline preparation-to-preparation variability in burst frequency was reasonable and similar between treatment groups. Had we evaluated the variability in burst-burst interval for slices (e.g., by calculating a regularity score), treatment with gabazine, or with combined gabazine and strychnine, which induced bursts of seizure-like activity, would likely have been shown to increase the variability in interburst interval (e.g., decrease burst regularity). By contrast to the frequency of bursting produced by slices, that produced by the island preparations used in this study tended to be somewhat more variable; whereas the slices from which islands were obtained burst at 0.2 – 0.5 Hz, the islands burst at 0.1 – 0.7 Hz. To minimize baseline island variability and the number of animals consumed to obtain island preparations we limited the islands used to only those bursting between 0.2 and 0.6 Hz. Given that the island preparation represents the most reduced preparation available for studying preBötC rhythmogenesis, it is perhaps not surprising that the frequency of bursting would be more variable in islands than in slices. In their initial description of the island preparation Johnson and colleagues [34] found that islands generated bursts at a higher frequency than slice preparations and that the SEM for preparation-to-preparation burst frequency was twice that in island preparations compared to slice preparations.

Although it did not do so in this study, using island preparations bursting over a wider range of baseline frequencies than slice preparations could contribute to a higher baseline frequency in islands than in slice preparations. However, such a difference would not, on its own, be likely to cause the differences in baseline burst frequency observed between island preparation treatment groups. Whereas islands used for testing the effects of NCPA in standard aCSF (Fig. 4A) burst at 0.37 ± 0.04 Hz, those used to examine the effects of NCPA in the presence of gabazine burst at 0.27 ± 0.04 Hz and those used to evaluate the effects of NCPA in the presence of gabazine and strychnine burst at 0.40 ± 0.06 Hz. This variability resulted from the distribution of baseline burst frequencies produced by island preparations in the various treatment groups. Whereas the burst frequencies generated by islands in the first (testing NCPA in standard aCSF) of these three groups were distributed fairly evenly between 0.2 and 0.6 Hz, those generated by islands in the second group (testing NCPA in the presence of gabazine) were distributed near the lower portion of the range with two of the islands bursting at the lower cutoff frequency. The frequency of bursts generated by island preparations in the third group (testing NCPA in the presence of gabazine and strychnine) clustered near the upper end of the allowed range with 2 of the preparations generating population-level bursts at the upper frequency limit. Although baseline firing frequency varied between island groups, the responses of those used to test the effects of NCPA and DPCPX in standard aCSF, or in aSCF containing gabazine were, as shown above, largely similar to those in the corresponding slice preparations. Both the increased baseline variability of islands and their responses to treatment may reflect the importance of modulatory input to preBötC neurons from other regions, such as the contralateral preBötC, and/or reduced intra-network communication.

Under normoxic baseline conditions it is unlikely that the preBötC would experience a substantial rise in extracellular adenosine concomitant with a substantial decrease in extracellular GABA and glycine concentration. However, hypoxic stress, after stimulating an initial augmentation of respiratory network activity, depresses respiratory network activity and decreases extracellular GABA levels [37] and glycinergic transmission [38] within the ventral respiratory group. These latter two effects are of interest since, as noted above, reducing GABA_A_ergic and glycinergic transmission within the neonatal respiratory network tend to increase network activity [29-31]. However, while hypoxia decreases GABA and glycine-mediated transmission, it also increases extracellular adenosine and serotonin levels, depresses extracellular glutamate levels [37] and alters a variety of membrane properties [39-43]. Although adenosine represents only one of numerous variables that contribute to hypoxic depression of respiratory network output, the data presented here verify that A_1_R activation is sufficient to overcome potential increases in network excitability caused by reduction in GABA and glycine transmission and in so doing depress preBötC bursting [*c.f*. [22-24,44]].

Throughout most of this study excitatory synaptic transmission between preBötC neurons was left intact so that population-level effects could be observed. By affecting presynaptic mechanisms of synaptic transmission, such as axon-terminal Ca^++ ^conductances, A_1_R activation can directly affect synaptic transmission [45,46]. In fact, our data show that NCPA decreased synaptic inputs to preBötC neurons. Thus, the data presented herein do not rule out the possibility that A_1_R activation may depress preBötC rhythmogenesis by directly inhibiting excitatory transmission between preBötC neurons. In fact, imunohistochemical data suggest that A_1_R are found at the axon terminals of interneurons within a variety of CNS regions, including the NTS where they may be involved in regulating transmitter release [47]. However, A_1_R activation clearly decreases excitability of preBötC neurons, an effect that alone can decrease transmitter release.

During the present study A_1_R activation decreased the R_in _of preBötC neurons regardless of whether or not those neurons were synaptically isolated from the rest of the network. Although not a quantitative measure of neuronal excitability due to its reliance on access resistance and seal resistance, holding current can reflect changes in membrane voltage that would occur, were the neuron not being subjected to voltage clamp. During the present study holding current increased (became more positive) in ~60% of the NCPA-treated neurons examined in synaptic isolation. By contrast, the holding current of control neurons (those examined in low Ca^++^/High Mg^++ ^aCSF with TTX, but without NCPA) became more negative over time. In brainstem-spinal cord preparations Herlenius and Lagercrantz found that A_1_R activation decreased the V_m _of expiratory neurons but did not affect R_in _or V_m _of inspiratory neurons [23]. The difference between their study and the data presented herein may reflect the types of neurons from which data were obtained. Whereas Herlenius and Lagercrantz [23] defined inspiratory neurons in terms of discharge characteristics, here inspiratory neurons were defined as any that received a barrage of synaptic input during the population burst. Some neurons received concurrent barrages of EPSCs and IPSCs resulting in little or no net inward/outward current, suggesting that although defined as inspiratory per the criteria used herein, these may have actually been expiratory neurons.

Although NCPA affected resting membrane properties in this study, it did not affect whole-cell currents evoked by depolarizing voltage steps. However, depolarizing voltage steps activate multiple conductances in preBötC neurons, and different types of inspiration-related neuron express different combinations of voltage-sensitive ion channels [12]. In other neurons A_1_R activation affects Ca^++ ^conductance [17,25,48]. It is possible that one or more of types of these conductances were affected by A_1_R, but in combination with whole cell K^+ ^conductances such changes were insufficient to affect total transmembrane current. Although beyond the scope of the present study, future work will provide a more detailed dissection of the effects of A_1_R activation on various membrane conductances. Rhythmogenesis within the preBötC of neonatal mice is thought to require synaptic interactions and the activity of pacemaker neurons [3,5,9,10,12,32,34,49-53]. Upcoming research in our laboratory will examine whether A_1_R activation decreases the excitability and rhythmic production of action potential bursts by synaptically-isolated preBötC pacemaker neurons.

## Conclusion

In this study A_1_R activation depressed preBötC rhythmogenesis by acting directly on the preBötC within slice and island preparations, even though the frequency of synaptic currents in preBötC neurons is extensively reduced in the latter preparation. Moreover, A_1_R-mediated depression of preBötC rhythmogenesis was similar in slices bathed in standard aCSF, in slices and islands bathed in aCSF containing gabazine, and in slices bathed in gabazine and strychnine. Even when chemical communication between preBötC neurons and other cells within the tissue was blocked, A_1_R activation affected resting membrane properties of preBötC neurons in a manner consistent with decreasing neuronal excitability. Agonizing A_1_R with NCPA decreased the frequency of synaptic inputs to preBötC neurons in both types of preparation. Together these data support the notion that, A_1_R-medated depression of preBötC rhythmogenesis involves both decreased neuronal excitability and inhibition of chemical synaptic communication between preBötC neurons. Although postsynaptic currents and potentials resulting from GABA_A _and glycine receptor activation may integrate synergistically with the modulatory actions of A_1_R activation, the data herein suggest that their relative contribution to such depression is minor.

## Methods

### Isolation and Maintenance of in vitro Preparations

All procedures were carried out according to guidelines established by NIH and the National Research Counsel, and were approved by the Institutional Animal Care and Use Committee at Central Michigan University. Slices of mouse medulla oblongata were obtained from male and female Swiss-Webster mice (= 7 d old) that were decapitated at the C3/C4 vertebral level. The brainstem was isolated in ice-cold aCSF (in mM: 118 NaCl, 3 KCl, 1.5 CaCl_2_, 1 MgCl_2_, 25 NaHCO_3_, 1 NaH_2_PO_4_, and 30 D-Glucose) saturated with carbogen gas (95% O_2 _and 5% CO_2_). The cerebrum and cerebellum were dissected away and the isolated brainstem was glued to an agar block using cyanoacrylate glue. This mount was secured in a vibrating microtome with the rostro-caudal axis of the brainstem and spinal cord tilted such that the top of the preparation was slightly farther away from the face of the microtome than the lower portion (the axis of the tissue was oriented ~110° from the plane in which the microtome blade advanced). Serial sections (*ca*. 300 μM thick) were removed from the rostral surface to reveal the 4^th ^ventricle. Then, *ca*. 200 μm-thick sections were removed until the region containing the preBötC was revealed, recognized by the presence of the obex at the caudal closure of the fourth ventricle, the appearance of XII nerve tracts and the IO. At this level, a 600 μm-thick slice was removed and immediately transferred to a recording chamber.

Slice viability was sustained by recirculating carbogen-saturated aCSF (29.5°–30.5°C; pH 7.4) between a reservoir and the recording chamber (200 ml total volume). Thirty minutes before baseline recording, the potassium concentration of the aCSF was elevated from 3 mM to 8 mM. Slices were subjected to each experimental condition for 20 to 30 minutes. Only slices that generated bursts at frequencies between 0.2 and 0.5 Hz were used in this study.

### Island preparations

Transverse medullary slices were reduced to island preparations as described by Johnson *et al*. [34]. To summarize, following baseline recordings regions of the slice immediately surrounding the preBötC were cut away using micro-iridectomy scissors. Cuts were made from the ventral margin of the slice adjacent (lateral) to the IO along a curve following and slightly lateral to the XII nerve tract. Then from a point ~1/3 of the way between the ventral fissure and the dorsal cusp of the fourth ventricle the next cut progressed laterally to the medial margin of SP5. The final cut then progressed along the ventromedial margin of SP5 to the ventrolateral surface of the slice, thereby removing the contralateral preBötC, NTS, SP5, XIIn, XII tract, and the IO (*c.f*. Johnson *et al*. 2001). The frequency at which islands generated population bursts was somewhat more variable than that observed in slice preparations. Accordingly, only islands that generated bursts of integrated network activity at frequencies between 0.2 and 0.6 Hz were used in this study.

### Extracellular recordings

Extracellular electrodes were fabricated from borosilicate glass pipettes, filled with aCSF, and connected to a homemade AC-coupled pre-amplifier (100 times amplification). Raw traces were filtered between (0.3–3 kHz) and amplified an additional 100 times (Amplifier model P15, Grass Technologies, West Warwick, RI, USA) before being sent to a hardware integrator (50 ms time constant) and an analog-digital converter (ITC-18, Instrutech Corp., Port Washington, NY, USA). Both raw and integrated traces were recorded on the hard disk of a personal computer using Chart 4.0 (ADInstruments, Inc., Colorado Springs, CO, USA) or PatchMaster v2.11 (HEKA Instruments, Inc., Southboro, MA, USA).

### Whole-cell patch clamp recordings

Whole-cell patch clamp recordings were obtained using unpolished electrodes fabricated from thick-walled borosilicate glass (Warner Instruments, # GC 150-10) and filled with (in mM) 140 K-Gluconate, 1 CaCl_2_, 2 MgCl_2_, 4 Na_2_ATP, 10 EGTA, and 10 HEPES (pH 7.2). Using near-infrared Normarski optics (with a 40× objective) the tip of the patch electrode was positioned on the soma of a neuron within the preBötC. After a gigaohm seal was established, whole-cell configuration was established by applying repetitive pulses of negative pressure until the cell membrane within the electrode tip ruptured. Transmembrane currents were recorded using an EPC8 amplifier (HEKA Instruments, Inc.) and recorded on the hard disk of a personal computer via Patchmaster software and an ITC-18 data acquisition board. Currents were filtered at 2 KHz using the internal Bessel filter of the amplifier and digitized at 10 kHz. Before recording any data from a cell, transient currents due to electrode and cell resistance and capacitance were minimized and serial resistance was 80% compensated. Recordings were corrected offline for a 15 mV junction potential. With the solutions used herein, Cl^- ^based spontaneous inhibitory postsynaptic currents (sIPSCs) appeared as outward cation currents (i.e., as an influx of Cl^-^) in cells voltage clamped at -35 mV, while those triggered by excitatory neurotransmitters appeared as inward currents (sEPSCs). To track the quality of the recording, we monitored fundamental properties including input resistance (R_in_), access resistance (R_a_), holding current at -60 mV (I_hold_) and cell capacitance throughout baseline and experimental conditions. Recordings in which R_a _became greater than 10% of R_in _were discarded as were any in which holding current at -60 mV exceeded (became more negative than) -400 pA. Whereas R_a _and cell capacitance were monitored using the manual adjustments on the amplifier, I _hold _was recorded directly from traces and R_in _was calculated based on the current observed (without leak subtraction) during a 20 ms-long voltage step from a holding potential of -60 mV to a command potential of -80 mV. Voltage-gated sodium currents (I_Na_) and steady state voltage gated potassium currents (I_Kd_) were evaluated using a voltage step protocol. From a holding potential of -60 mV we applied 200 ms long voltage steps from -80 to 20 mV in 10 mV steps. Linear leak currents were eliminated with an online P/4 leak subtraction protocol.

In a subset of voltage clamp experiments, the effects of NCPA on R_in _and I_Kd _were evaluated using synaptically-isolated inspiratory neurons. Isolation was accomplished by bathing slices in aCSF lacking (severely reduced) Ca^++^, and containing Tetrodotoxin (TTX; 1 μM), CdCl_2 _(200 μM), and MgCl_2 _(10 mM).

### Solutions

All drugs/toxins were applied by diluting stock solutions 1000 times in the recirculating bath reservoir. The concentrations listed represent the final working concentration for each agent. The adenosine A_1_-receptor agonist N^6^-Cyclopentyl Adenosine (NCPA; 1 μM), the adenosine A_1_-receptor antagonist 1,3-Dipropyl-8-cyclopentylxanthine (DPCPX; 1 μM), the GABA_A_-receptor antagonists bicuculline (20 μM, free base) and gabazine (20 μM) were prepared as stock solutions in DMSO. The glycine receptor antagonist strychnine (1 μM) and TTX were prepared as stock solutions in deionized water.

### Data analysis

Throughout this report, the term baseline is used in reference to measurements performed prior to addition of drugs. The term control is used to refer to recording in aCSF without drug after a period intended to match that of drug application in a separate preparation. Extracellular data (burst frequency and amplitude) were measured using population bursts occurring during the final 2-minutes of each treatment (Igor Pro 4.07, Wavemetrics Inc., Oswego, OR). All data were tested for normality (Minitab v. 14, Minitab, Inc., State College, PA, USA). Extracellular data having a normal distribution were analyzed using repeated measures ANOVA followed by Tukey post-hoc comparisons to determine differences between specific treatments, when appropriate. Non-normal extracellular data were evaluated using Friedman's Repeated Measures ANOVA on ranks. Again differences between specific treatments were evaluated with Tukey post-hoc tests. Normally distributed intracellular data were compared using paired-t tests for comparisons within a cell, or two-sample t-tests when comparing cells from separate treatments. Non-normal intracellular data were compared using the Mann-Whitney Rank Sum test. Differences were considered significant at P ≤ 0.05. Data are presented as means ± SE.

## Authors' contributions

RJV performed most of the extracellular recordings from slice and island preparations, acquired some of the voltage clamp recording, and helped draft the manuscript. EJS conducted some extracellular recordings from slice preparations, most of the voltage clamp recordings, and provided critical feedback during manuscript preparation. JDK conceived of the study, designed the experiments, performed some of the extracellular recordings from slice preparations, carried out the statistical analyses, and was the primary writer of the manuscript. All authors read and approved the final manuscript.
